# Synergistic effect of GF9 and streptomycin on relieving gram-negative bacteria-induced sepsis

**DOI:** 10.3389/fbioe.2022.973588

**Published:** 2022-08-30

**Authors:** Bing Wei, Yingmin Ma

**Affiliations:** ^1^ Emergency Medicine Clinical Research Center, Beijing Chao-Yang Hospital, Capital Medical University, and Beijing Key Laboratory of Cardiopulmonary Cerebral Resuscitation, Clinical Center for Medicine in Acute Infection, Capital Medical University, Beijing, China; ^2^ Department of Respiratory and Critical Care Medicine, Beijing Youan Hospital, Capital Medical University, Beijing, China

**Keywords:** nanomedicine, GF9, streptomycin, sepsis, Gram-negative bacteria-induced

## Abstract

Triggering receptor expressed on myeloid cells-1 (TREM-1) regulates inflammation and promotes a vigorous immune response. GF9 is one of the peptides that inhibit the mTREM-1 signaling pathway, thus reducing the inflammatory mediators in diseases including sepsis. Nanotechnology could offer a new complementary strategy for diseases. Streptomycin is also one treatment of sepsis. However, the role of nanoparticles delivered GF9 combined with streptomycin on sepsis had never been discovered. In the present study, cecal ligation and puncture (CLP) and lipopolysaccharide [LPS, *Escherichia coli* (*E. coli*) O111:B4] sepsis models were constructed. SDS-PAGE was used to evaluate the size of nano drugs; Western blot was used to detect the protein levels of MMP2 and TREM-1 in cells. The levels of TNF-α and IL-6 were detected by ELISA. Histopathological changes were observed by HE staining. And the nanomedicines of GF9-HFn/Str were successfully constructed. The size of GF9-HFn/Str is 40 kD. The ferritin-based nanoparticle plays a vital role in delivering streptomycin into cells and tissues. GF9 (1.6 μM) and streptomycin (40 μM) co-delivery nanomedicine showed a better effect on promoting overall survival, decreasing *E. coli*, significantly suppressed the expression levels of inflammatory factors (TNF-α and IL-6), and can reduce lung injury. Our study demonstrated that combination delivery of nanomedicine GF9 and streptomycin have a better effect on overall survival rate, anti-inflammatory, and anti-bacterial in sepsis. Our present study revealed a new potential therapeutic method for sepsis.

## Introduction

Sepsis is a common complication after severe burns, trauma, infection, shock, and surgery, leading to septic shock and multiple organ dysfunction syndromes (Gyawali et al., 2019). Sepsis is mainly caused by pathogenic bacteria infection, with Gram-negative bacteria *Escherichia coli* (*E. coli*) accounting for 24.78% (Zou H. et al., 2021). Measures as early anti-infection treatment, fluid resuscitation, and vasoactive drugs are mainly adopted for sepsis treatment (Meng et al., 2018). However, these treatments were not effective enough, and the sepsis-related mortality toll remains substantial. Therefore, new therapies were urgently needed.

With the in-depth exploration of the micro-world, nanomaterial was a new complementary strategy for diseases (Lippert and Zachos, 2007). Nanoparticles are under development for an increasing number of applications. Common natural bio-nanoparticles mainly include liposome protein, heat shock protein, ferritin, and viruses (Stanley, 2014). Among genetically engineered nanoparticles, ferritin is the best characterized in the field of bio nanotechnology (Kim et al., 2021). Ferritin nanoparticles were characterized as good biocompatibility, large drug-carrying capacity, and biodegradability. Ferritin nanoparticles have been proven to be a powerful platform for antigen presentation and drug delivery (Khoshnejad et al., 2018; Bellini et al., 2020). The nanomedicines were made by connecting particular small molecule medicine, matrix metalloproteinase (MMP) substrates, and ferritin protein shells onto the outer surface of nanoparticles. Then, the nanomedicine was delivered into cells, cleaved and activated by MMPs. Finally, the medicine was released and played its role in disease. For example, Zhen et al. (2013) used ferritin to encapsulate doxorubicin as a drug delivery vehicle. Engineered ferritin nanoparticles for the bioluminescence tracking of nanomedicine delivery in cancer (Bellini et al., 2020). Nanomedicine was widely used in cancer studies. However, few studies of ferritin-based nanomedicine were used in sepsis.

Triggering receptor expressed on myeloid cells-1 (TREM-1) regulates inflammation, leads to immune response (Gibot et al., 2019), and is a new biomarker for sepsis (Su et al., 2012). In associated inflammatory diseases, TREM-1 is highly expressed on inflammatory cells such as neutrophils, monocytes, and macrophages, and promotes the secretion of inflammatory factors and induces the production of pro-inflammatory factors, tumor necrosis factors and chemokines (Saurer et al., 2017; Peng et al., 2019). GF9 is a peptide that inhibits TREM-1 through DNAX-activating protein 12 kD (DAP12), with a concomitant reduction of inflammatory mediators in sepsis (Dantas et al., 2020). Streptomycin is also one treatment of sepsis. However, the role of nanoparticles delivered GF9 combined with streptomycin on sepsis had never been discovered.

Based on previous studies, we hypothesized that the combined administration of nano drug GF9 and streptomycin could alleviate sepsis induced by gram-negative bacteria. We tested the hypothesis in CLP induced sepsis mouse model *in vivo* and LPS induced sepsis cell model *in vitro*.

## Materials and methods

### Nanomedicine preparation and evaluation

Hollow ferritin (HFn), streptomycin, and GF9 were bought from AmyJet Scientific Ltd. (Wuhan, China), Abcam (United States), and New England Peptide (Gardner, MA), respectively. The present study used the ferritin protein as a biological functional protein-based nanoparticle shell and bound molecules as streptomycin and GF9. Four ferritin delivery groups were prepared, including hollow ferritin (HFn), streptomycin delivery of hollow ferritin (HFn/Str), GF9 delivery of hollow ferritin (GF9-HFn), and GF9 and streptomycin co-delivery of hollow ferritin (GF9-HFn/Str).

The emulsification method, according to Li et al. (2019) was used to prepare nanomedicine. Briefly, GF9 (1.6 μM) and streptomycin (40 μM) diluted in CH_2_Cl_2_ were mixed as the oil phase, and HFn (1 mg/ml) dissolved in phosphate buffer saline (PBS) was used as the water phase. Then, the oil and water phases were mixed with 1:1 v/v and ultrasound in a mechanical ultrasonic instrument. After the organic solvent is completely evaporated, it is transferred to the dialysis tube, and dialyzed in ultrapure water overnight, replacing the water 2 h/time. Finally, the solution was freeze-dried for 36 h and re-dissolved in sterile water to obtain a concentrated nanomedicine experimental solution.

The prepared nanomedicines were imaged with a transmission electron microscope (TEM; JEM-1230; JEOL, Tokyo, Japan) at 60 Kv.

### The MMP2 response of GF9-HFn

MMP2 was obtained from Beyotime Biotechnology Co., Ltd. (Shanghai, China). Briefly, 0.4 mg of GF9-HFn was dissolved in 4 ml of PBS and divided into two groups, with one treated with MMP2 (GF9-HFn + MMP2 group) and the other treated without MMP2 (GF9-HFn group).

### *In vitro* and *in vivo* sepsis model construction

*In vivo* sepsis model was constructed using cecal ligation and puncture (CLP) method reported previously (Zhao et al., 2016). Firstly, the mice were anesthetized by isoflurane, local analgesine by 1% bupivacaine, and an incision was made in the midline. Secondly, the cecum was eviscerated, ligated using 5–0 silk suture, and punctured through-and-through using a 20-gauge needle. Thirdly, the abdomen was closed layer by layer. Finally, the mice in sham groups were manipulated in the same way, without the CLP component. All animal experiments were performed following the National Institutes of Health Guide for the Care and Use of Laboratory Animals.

For *in vitro* analysis, the Human umbilical vein endothelial cells (HUVECs) were obtained from the American Type Culture Collection (ATCC, Manassas, United States). They were cultured in dulbecco’s modified eagle medium (DMEM) (HyClone, Logan, Utah, United States) containing 10% fetal bovine serum (FBS) (Biological Industries, Israel) and 1% penicillin-streptomycin (HyClone, United States), and then cultured at 37°C, 5% CO_2_. The HUVECs were treated with lipopolysaccharide (LPS) (*E. coli* O111:B4, Sigma, St Louis, MO), HFn, GF9-HFn, HFn/Str, and GF9-HFn/Str.

### Sodium dodecyl sulfate-polyacrylamide gel electrophoresis and western blot analyses

We evaluated the size of nanomedicine by SDS-PAGE in nanomedicines of HFn, GF9, GF9-HFn, GF9-HFn + MMP2, and GF9-HFn/Str. SDS-PAGE was performed according to the method reported by Partikel et al. (2019). Briefly, the proteins were desorbed from the nanocage surface by shaking in 30 µl reducing loading buffer (Roti-Load®1) overnight. Before denaturing the proteins at 5 min at 95°C, the samples were centrifuged at 30,000 g for 45 min. Then, the SDS-PAGE was performed using 10% polyacrylamide gel and electrophoresis under 200 V for 1 h on an OmniPAGE mini system (Omnilab-Laborzentrum GmbH & Co. KG, Bremen, Germany). Finally, the gels were fixed, stained, destained, and observed under Gel ix Imager (INTAS Science Imaging Instruments GmbH).

The protein levels of MMP2 and TREM-1 were evaluated in control and LPS cells. The expression level of TREM-1 was assessed in sepsis patients and *in vivo* models (Control and sepsis). The proteins in cells and tissues were lysate in RIPA buffer (Thermo Fisher Scientific, Waltham, MA, United States) and quantified by a BCA protein assay kit (Invitrogen, Carlsbad, CA) referring to the instruction. Then, the proteins were separated by SDS-PAGE mentioned above and were transferred to poly (vinylidene fluoride) (PVDF) membranes. The membranes were blocked and incubated with primary antibodies of MMP2 (1:1,000; #ab37150; Abcam) and TREM-1 (1:200; #ab217161, Abcam) at 4°C over 12 h. The secondary antibody of horseradish peroxidase-conjugated goat anti-rabbit IgG (#ab6721; 1:5,000; Abcam) was incubated with the washed membranes for 1 h at 25°C. Finally, the bands were observed under chemiluminescence (Pierce; Thermo Fisher Scientific, Inc.).

### Enzyme-linked immunosorbent assay

The levels of tumor necrosis factor-α (TNF-α) and interleukin-6 (IL-6) were assessed *in vitro* and *in vivo* by ELISA. Before the nude mice were sacrificed, blood was taken from the eyes of each group of nude mice, stored in a 1.5 ml EP tube, and quickly centrifuged at 4°C at 3000 rpm for 15 min, and then the upper serum was taken into the EP tube. According to the manufacturer’s instructions, the experiments were performed using TNF-α and IL-6 ELISA kits purchased from Sigma-Aldrich (St. Louis, MO, United States).

### Streptomycin release

About 1 ml of a solution containing nanomedicines was put into a dialysis bag and added with PBS buffer (pH 7.4) containing 0.5% Tween 80. At 4°C and 37°C, the solutions were shaking at 100 r/min. Then, the accumulation of streptomycin was measured under a microplate reader. Each sample was tripled.

### Anti-bacterial evaluation

The *E. coli* proliferation was evaluated *in vitro* in groups of control, LPS, HFn, and HFn/Str. It was also assessed *in vivo* in tissues of liver, spleen, peritoneal lavage fluid (PLF), and blood in groups of CLP, HFn, HFn/Str, GF9-HFn, and GF9-HFn/Str. The antibacterial activity of the nanoparticles was tested against bacteria commonly found on wounds: *E. coli* (Gram negative: ATCC 25922). Luria Bertani agar (Merck, Germany) was used for the cultivation of bacteria. The initial bacteria concentration of *E. coli* colony-forming units (CFU) was calculated, placed in the middle of the culture, and incubated at 37°C for 24 h. Finally, the bacteria colony were photographed.

### Flow cytometric analysis of fluorescein isothiocyanate-labeled streptomycin

FITC (24 μmol) was dissolved in 10 ml of acetone. The nanomedicines were dissolved in 10 ml of PBS, added to the reaction flask, and stirred for 2 days in darkness at room temperature (Kolhe et al., 2003). The crude product was purified by dialysis against deionized water and recovered by lyophilization. The nanomedicine with streptomycin was stained with streptomycin-FITC (BD Biosciences) in FACS buffer (1% FBS in PBS) for 20 min. After the experiment, the liver, kidney, spleen, and lung tissues were isolated and crushed. The cell suspension was collected and analyzed by one flow cytometric (CyFlow Ploidy Analyzer, Partec, GMBh, Germany).

### Hematoxylin and Eosin staining

The lung tissues were isolated from mice in groups of CLP, HFn, HFn/Str, GF9-HFn, and GF9-HFn/Str. The tissues were fixed in 10% neutral formaldehyde solution for 24 h, dehydrated, transparent, and waxed. Then, we cut the paraffin-embedded tissues into 4 μm thick sections. Finally, the sections were stained by hematoxylin and eosin (H&E), dehydrated, and observed under a 200× microscope.

### Pharmacokinetics analysis

The pharmacokinetics of Str, HFn/Str, and GF9-HFn/Str in plasma samples of mice were evaluated for total doxorubicin (DOXO). The DOXO was measured using the flow cytometry method.

### Quantitative real-time polymerase chain reaction

TRIzol® reagent (Invitrogen; Thermo Fisher Scientific, Inc.) was applied for cell RNA extraction. The relative expression levels of antimicrobial peptides (AMPs) of hepcidin and LL-37 were evaluated in nanomedicines of control, LPS, HFn, and HFn/Str. The relative expression of hepcidin and LL-37 were measured using the 2^−ΔΔCt^ method (Livak and Schmittgen, 2001), using glyceraldehyde-3-phosphate dehydrogenase (*GAPDH*) as the reference. The primers are listed as Table 1. The primers were synthesized by Genscript (Nanjing, China). Then, one-step qRT-PCR kits obtained from Vazyme Biotech Co. Ltd. (#Q221-01; China) were applied for qRT-PCR analyses. The PCR productions were finally conducted on the 7300 RT-PCR system (Foster City, CA).

**TABLE 1:** Sequence of primers used for RT-PCR.

Gene name	Forward primer (5′-3′)	Reverse primer (5′-3′)
hepcidin	GAA​GGC​AAG​ATG​GCA​CTA​AGC​A	TCT​CGT​CTG​TTG​CCG​GAG​ATA​G
LL-37	TAA​CCT​CTA​CCG​CCT​CCT​GGA​CCT​GGA​CC	GGA​CTC​TGT​CCT​GGG​TAC​AAG​ATT​CCG​C-3
GAPDH	GTC​AGT​GGT​GGA​CCT​GAC​CT	AGG​GGT​CTA​CAT​GGC​AAC​TG

### Statistical analysis

The statistical analyses and graph drawing were carried out using GraphPad Prism 8.0 software (San Diego, CA, United States). Data are presented as mean ± standard deviation (SD). The overall survival rate was analyzed by Kaplan Meier analysis and the log-rank test. Student’s t-test and one-way ANOVA were performed to analyze data between two groups and more groups. *p* < 0.05 considered as statistically significant different.

## Results

### The evaluation of ferritin-based co-delivery nanoparticles

**FIGURE 1:**
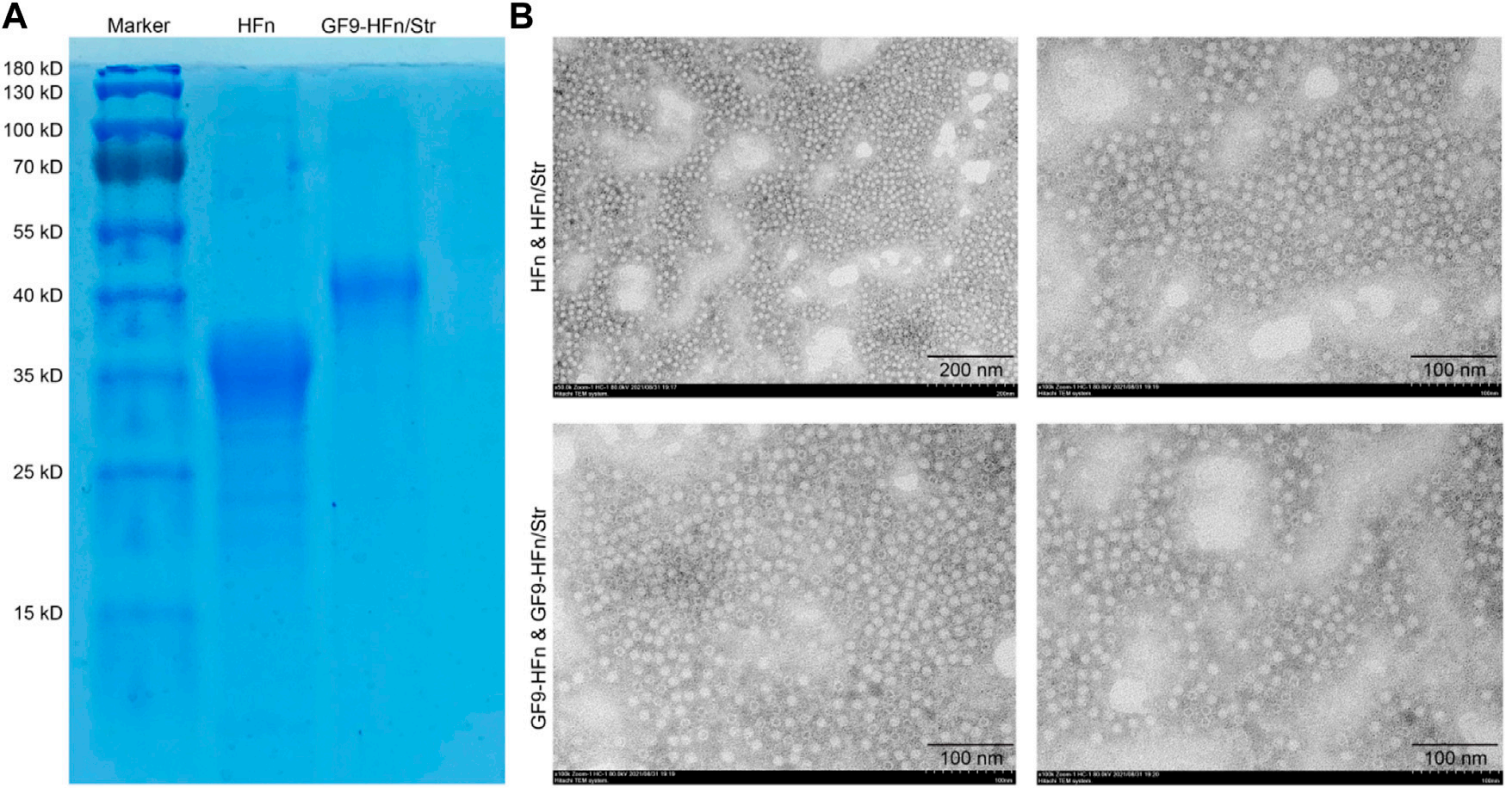
The evaluation of ferritin-based co-delivery nanomedicine. **(A)** SDS-PAGE evaluated the size of ferritin-based nanomedicine. **(B)** The morphology of nanomedicine was observed under TEM. SDS-PAGE, sodium dodecyl sulfate-polyacrylamide gel electrophoresis. TEM, transmission electron microscope.

The successfully constructed molecule delivery nanoparticles were the basis of the present study. The nanomedicines were evaluated in size and morphology. We found that the size of GF9-HFn/Str (40 kD) is bigger than HFn (35 kD; Figure 1A). We can observe from the TEM that the nanoparticles carrier is spherical, and the particle size distribution is relatively uniform. The ferritin-based nanoparticles can be seen in GF9-HFn and GF9-HFn/Str groups (Figure 1B). These results revealed successfully constructed nanomedicines for further study.

### GF9 can be released by MMP2

**FIGURE 2:**
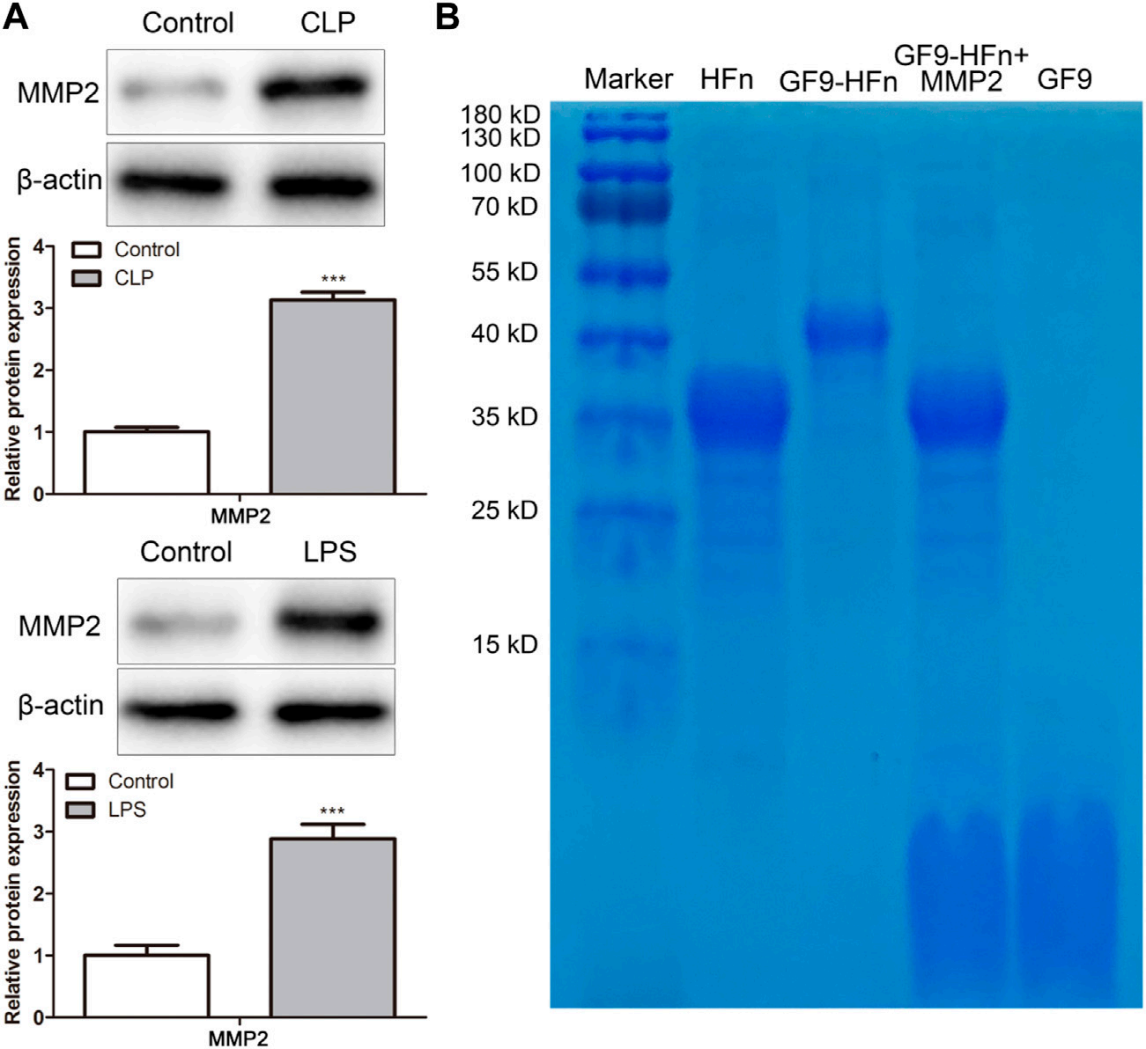
GF9 can be released by MMP2. **(A)** The protein expression level of MMP2 was assessed *in vivo* and *in vitro*. **(B)** The size of nanomedicines was assessed by western blot. MMP2, matrix metalloproteinase-2. ***p < 0.001 *vs*. Control.

In order to evaluate the response of MMP2 enzyme stimulation to nanomedicine, we firstly assessed the protein expression of MMP2 *in vitro* and *in vivo*. The western blot result showed that MMP2 expression level was significantly increased *in vitro* and *in vivo* compared with control (*p* < 0.01; Figure 2A). Then, SDS-PAGE assessed the protein molecules. Compared with GF9-HFn (40 kD), the cells added MMP2 enzyme separated the band of GF9-HFn + MMP2 (35 kD) into two bands, which are HFn (35 kD) and GF9 (2.9 kD) (Figure 2B). The result revealed that the MMP2 enzyme could digest GF9, and the nanomedicine we prepared can enter into the cells and tissues, thus releasing GF9.

### Anti-inflammatory role of nanomedicine

**FIGURE 3:**
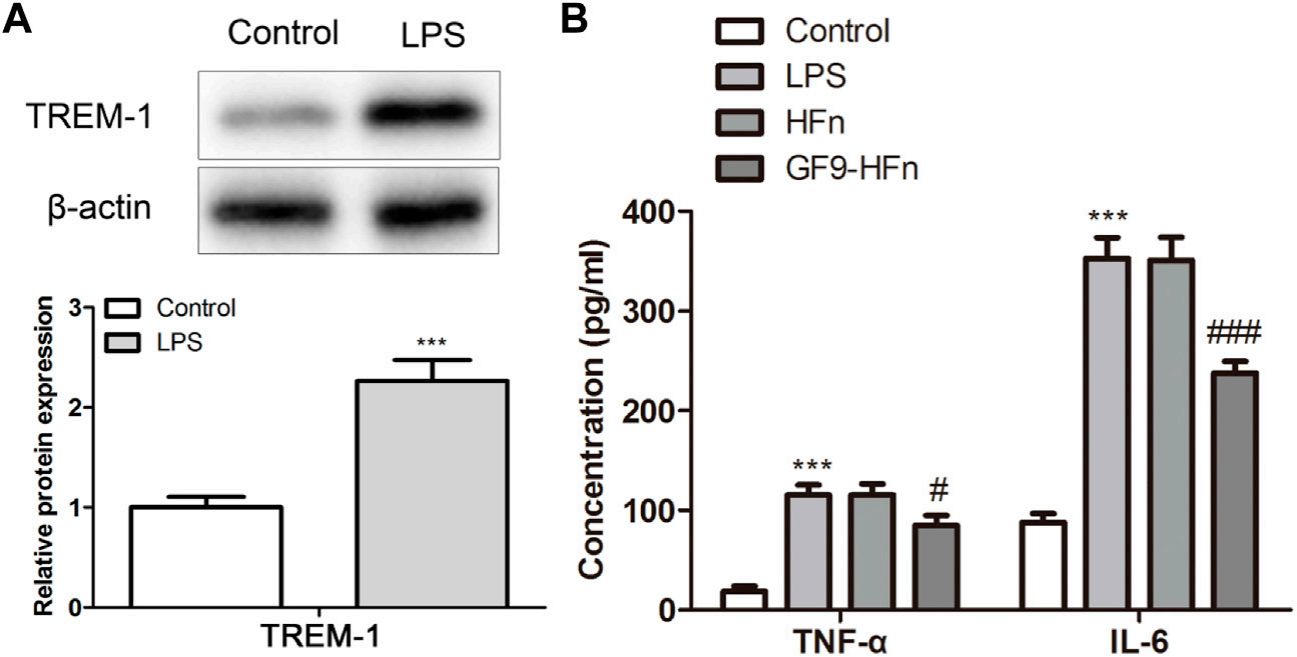
The inflammatory analysis of nanomedicines. **(A)** The expression level of TREM-1 was analyzed using a western blot. **(B)** The levels of inflammatory cytokines of TNF-α and IL-6 were evaluated. TREM-1, triggering receptor expressed on myeloid cells-1. TNF-α, tumor necrosis factor-α. IL-6, interleukin-6. ***p < 0.001 *vs*. Control. ^#^ *p* < 0.05, ^###^ *p* < 0.001 *vs*. Control.

The expression of TREM-1 was assessed by western blot. The protein expression of TREM-1 was higher in the LPS-induced sepsis *in vitro* model than in the control (*p* < 0.01; Figure 3A). In addition, the expression levels of inflammatory cytokines as TNF-α and IL-6 were assessed by ELISA. The results showed that compared with control group, TNF in LPS group-α And IL-6 expression was significantly increased; Compared with HFN group, TNF in GF9 HFN group-α And IL-6 expression was significantly reduced (*p* < 0.01; Figure 3B). These results revealed that nanomedicine of GF9-HFn significantly reduces the expression level of inflammatory cytokines.

### Anti-bacterial role of nanomedicine

**FIGURE 4:**
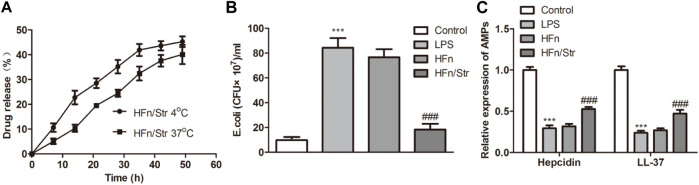
The effect of nanomedicines on anti-bacterial. **(A)** The streptomycin release analysis by nanomedicines at 4°C and 37°C. **(B)** The levels of *E. coli* in nanomedicines were measured. **(C)** The expression level of AMPs in nanomedicines. ***p < 0.001 *vs*. Control. ^###^ *p* < 0.001 *vs*. Control.

The anti-bacterial of nanomedicine was evaluated by testing the content of streptomycin released at both 4°C and 37°C. At the first 20 h, the consent of streptomycin released at 4°C promoted a faster release than at 37°C (Figure 4A). While the release amount gradually converged at 50 h, which is about 42%. The *E. coli* amount was significantly reduced by HFn/Str compared with HFn (*p* < 0.01; Figure 4B). In addition, compared with HFn group, the expression of hepcidin and LL-37 in HFn/Str group was significantly upregulated (*p* < 0.01; Figure 4C). These results indicated a promising antibacterial activity of nanomedicine on sepsis.

### Ferritin-based nanoparticle deliver streptomycin into cells and tissues

**FIGURE 5:**
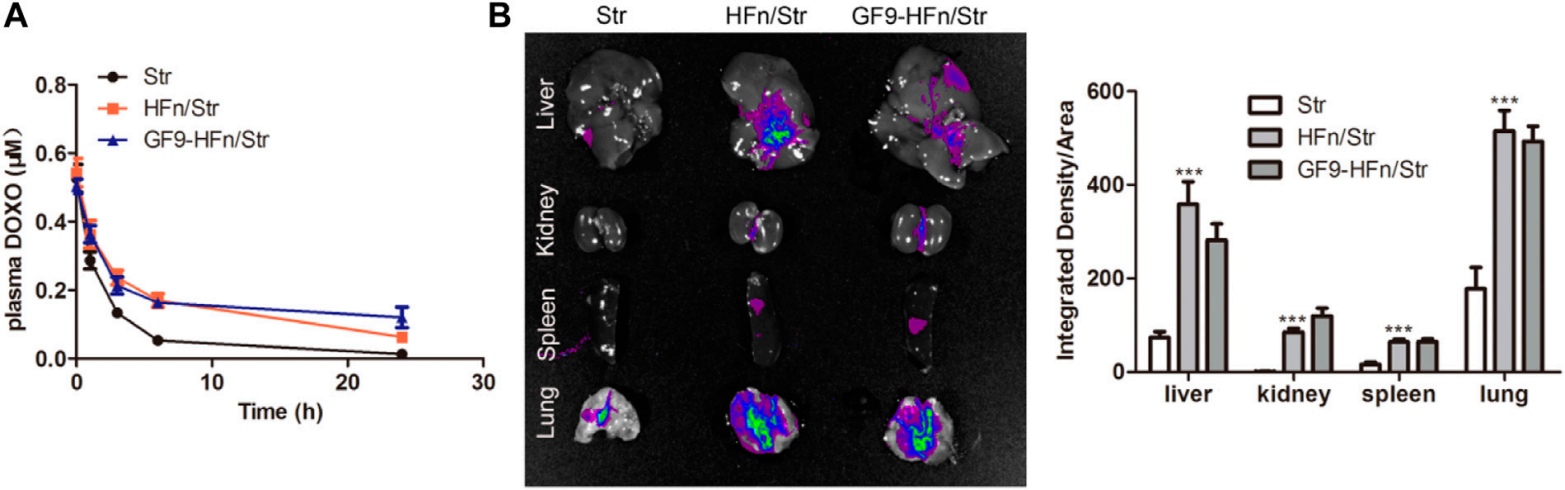
Pharmacokinetics analysis on nanomedicines. **(A)** The serum pharmacokinetics analysis on Str, HFn/Str, and GF9-HFn/Str. **(B)** The distribution of streptomycin in tissues. ***p < 0.001 *vs*. Str.

Pharmacokinetics was performed to study the transport process of the drug in the serum in nanomedicine. As a result, the serum concentration of FITC-streptomycin in HFn/Str and GF9-HFn/Str was higher than that in Str (Figure 5A). Moreover, the FITC-streptomycin co-delivered with GF9 has a higher FITC expression level in the tissues of the liver, kidney, spleen, and lung (Figure 5B). These results confirmed that the ferritin-based nanoparticle plays an essential role in delivering streptomycin into cells and tissues, which provided a basis for the following experiments.

### TREM-1 was highly expressed in bacterial-induced sepsis

**FIGURE 6:**
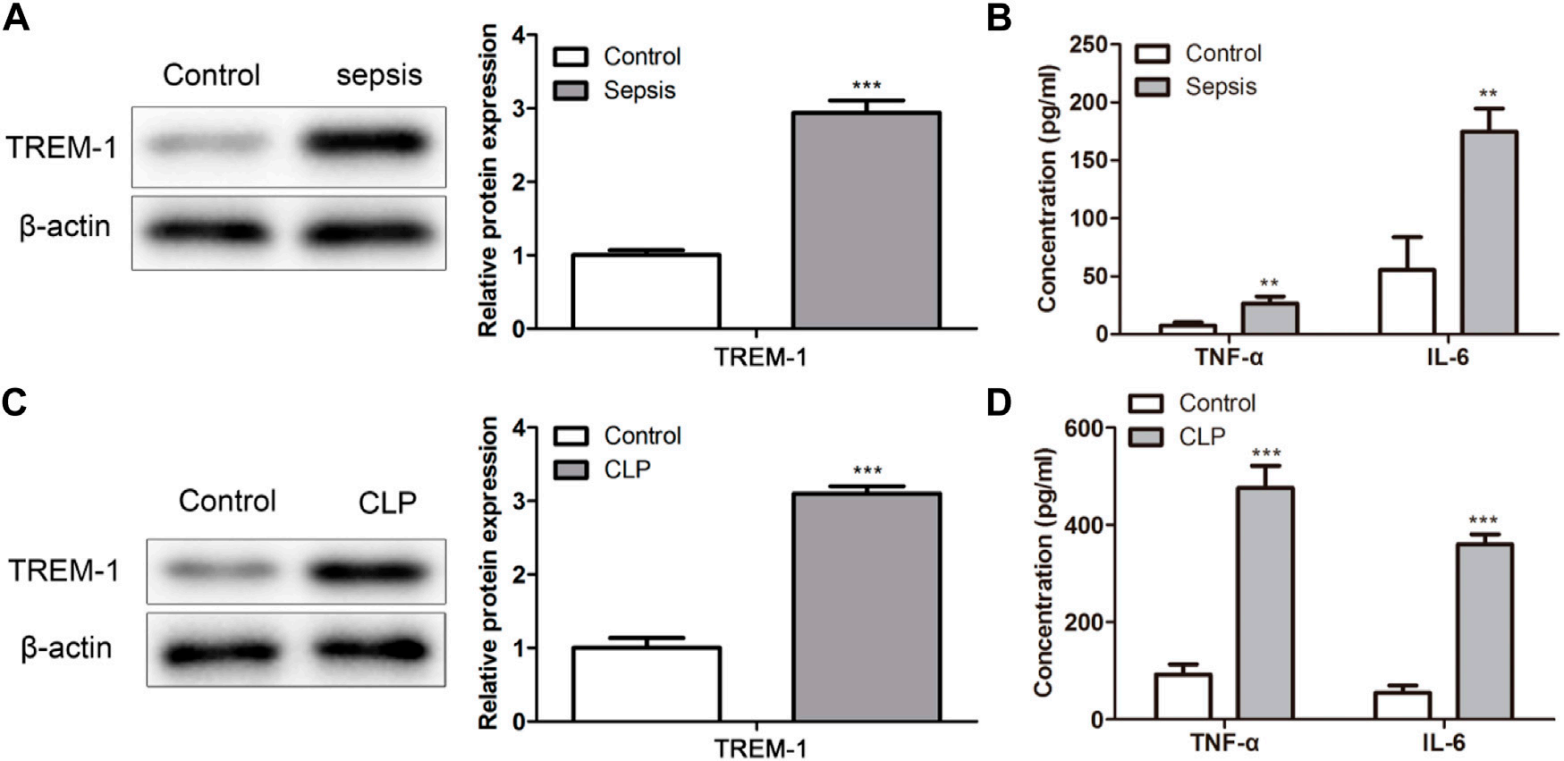
TREM-1 was highly expressed in bacterial-induced sepsis *in vitro*. **(A,C)** The TREM-1 expression level in sepsis was evaluated in sepsis patients and mice. **(B,D)** The levels of TNF-α and IL-6 were assessed in sepsis patients and mice. **p < 0.01, ***p < 0.001 *vs*. Control.

To evaluate the TREM-1 expression level in sepsis, the clinical patients and *in vivo* models with sepsis were used. TREM-1 was significantly highly expressed in bacterial-induced sepsis patients (*p* < 0.01; Figure 6A). Moreover, the inflammatory cytokines of TNF-α and IL-6 were significantly higher in sepsis patients than in control (*p* < 0.01; Figure 6B). The expression level of TREM-1, TNF-α, and IL-6 in the sepsis model showed a consistent expression trend in sepsis patients (Figures 6C,D). These results demonstrated that TREM-1 was highly expressed in bacterial-induced sepsis, and inflammatory was induced in sepsis.

### GF9 and streptomycin co-delivery nanomedicine relieves *E. coli*-induced sepsis

**FIGURE 7:**
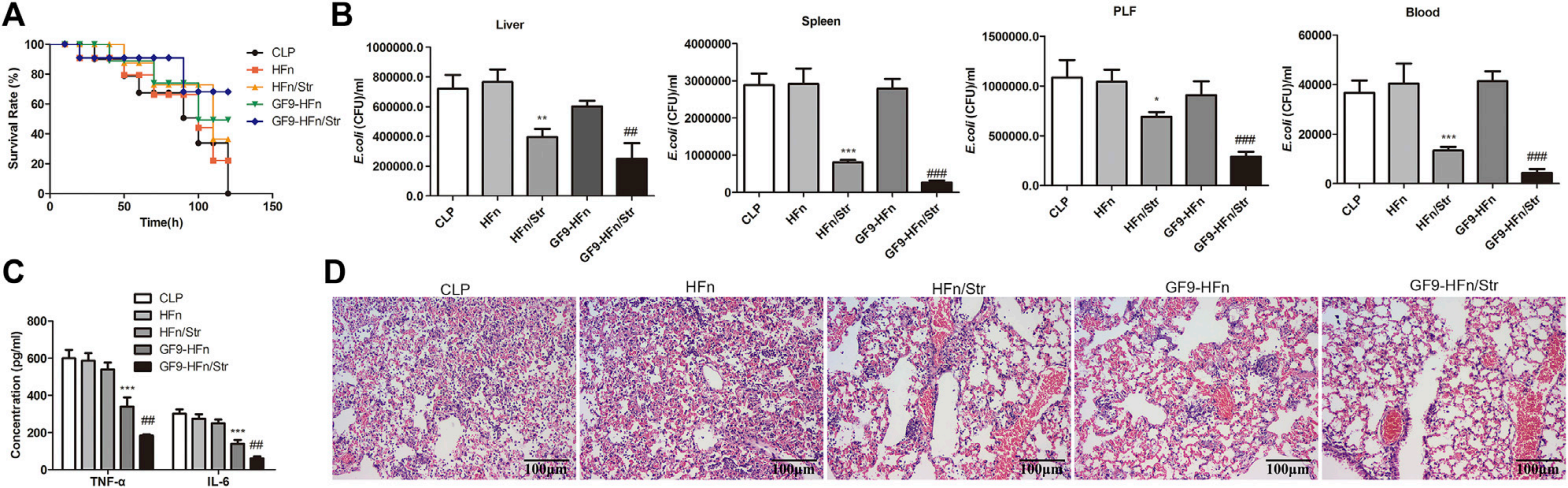
GF9 and streptomycin co-delivery nanomedicine relieve *E. coli*-induced sepsis. **(A)** The overall survival of mice was calculated. **(B)** The levels of *E. coli* in nanomedicines were measured in different tissues *in vivo*. **(C)** The levels of TNF-α and IL-6 were assessed *in vivo*. **(D)** The Hematoxylin and eosin (H&E) staining evaluated the lung damage of sepsis by nanomedicines. *p < 0.01, **p < 0.01, ***p < 0.001 *vs*. CLP. ^##^ *p* < 0.01, ***p < 0.001 *vs*. CLP.

Finally, we assessed the role of nanomedicines on sepsis *in vivo*. GF9-HFn/Str significantly promoted the overall survival rate compared with GF9-HFn and HFn/Str (*p* < 0.01; Figure 7A). Moreover, GF9-HFn and HFn/Str exhibited a higher overall survival rate than HFn and CLP *in vivo* (Figure 7A). The *E. coli* was significantly decreased by GF9-HFn/Str and HFn/Str compared with CLP, HFn, and GF9-HFn (*p* < 0.01; Figure 7B). Compared with the expression levels of TNF-α and IL-6 in CLP, HFn, and HFn/Str, they were significantly reduced in GF9-HFn/Str, followed by GF9-HFn (Figure 7C). As observed, the lung tissues in CLP and HFn were severely injured in sepsis mice *in vivo*. In mice injected with GF9-HFn/Str, the cells in lung tissue were regularly distributed, and only slight damage was observed (Figure 7D). Combined, we concluded that GF9 and streptomycin co-delivery nanomedicine promotes overall survival rate, decreases *E. coli*, represses inflammation, and reduces cell injury.

## Discussion

In recent years, the nanomedicine delivery system has been widely used in the treatment of diseases. However, some delivery drugs cannot penetrate deep into the target tissues for the structural disorder and high interstitial pressure (Liang et al., 2022). Therefore, it is essential to construct a drug delivery system with disease targeting and internal penetration ability. In the present study, we constructed a ferritin-based nanomedicine delivery system to transport GF9 and streptomycin into cells and tissues. This study found that co-delivery has a better effect than single treatment on improving overall survival, decreasing inflammatory and bacterial, and reducing lung cell damage.

In recent years, ferritin-based nanomedicine systems have been used and played essential roles in cancer therapy (Lee et al., 2017; Pandolfi et al., 2017). Pandolfi et al. (2017) analyzed the effect of nano-drug H-ferritin-curcumin on triple-negative breast cancer and found that the nanomedicine can enhance the cytotoxic effect of doxorubicin by interfering with the activity of multidrug resistance transporters; block MDA-MB-231 in G0/G1 phase, and accumulate MDA-MB-468 in G2/M phase. Inoue et al. (2021) demonstrated that doxorubicin-loaded ferritin heavy chain could be taken up and induce apoptosis of cancer cells overexpressing TfR1. Moreover, they also demonstrated that ferritin nanocage could deliver more than ten drug molecule types. This result supported our co-delivery assumption in the present study. We constructed a co-delivery nano-drug system by adopting both GF9 and streptomycin on H-ferritin nanocage in the present study. We tested the co-delivery nanomedicine in size and morphology by SDS-PAGE and TEM, and demonstrated that the co-delivery nanomedicine might be successfully constructed.

Then, the nanomedicine microenvironment was evaluated. The stimulus drug delivery system is an intelligent drug delivery system that can cause corresponding physical or chemical changes in the microenvironment to achieve controlled release of drugs (Zou T. et al., 2021). Currently, many smart nanomedicines that respond to external stimuli, such as light, magnetic field, and ultrasound, as well as internal stimuli, such as pH, temperature, enzymes, and redox potential reactions, have been explored (Zhou et al., 2018; Li J. et al., 2021). Some special enzymes will change under pathological conditions, such as inflammation or injured tissues (Fang et al., 2019; Ibraheem et al., 2019). These changes achieve enzyme-mediated drug delivery by stimulating responsive drug delivery systems, thereby delivering the drug to the target site. MMPs, especially MMP2, were reported to be upregulated in various diseases. Its substrate can be used to connect ferritin protein shell and molecule drugs and then connect to the surface of the nanocage, thus stimulating the nanomedicine system (Lee et al., 2015; Xu et al., 2019). Otherwise, MMP2 was demonstrated to be a key regulator in sepsis (Li B. et al., 2021). Xu et al. (2019) revealed a combined chemotherapy and photodynamic therapy by transporting the MMP2-triggered nanodrugs into the tumor sites. Lee et al. (2015) used MMP2 and constructed a double-chambered protein nanocage loaded with γ-carboxyglutamic acid of protein C (PC-Gla) and thrombin receptor agonist peptide (TRAP) for sepsis treatment. In the present study, we found that the expression of MMP2 was upregulated in sepsis *in vivo* and *in vitro*. Moreover, the nano-drug of GF9 can be digested by MMP2 enzyme. The MMP2 response analysis revealed that we constructed a successful ferritin-based nanomedicine delivery system by GF9 and streptomycin.

As reported, TREM-1 are upregulated in sepsis to severe as mediators of inflammation (Denning et al., 2020). As the inhibitory peptide of TREM-1, GF9 attenuated TREM-1 expression and diminished production of proinflammatory cytokines, resulting in decreased steatosis and tissue damage in the liver (Dantas et al., 2020). These results were consistent with that reported in our present study. We demonstrated that TREM-1 was upregulated in sepsis patients and *in vivo* models. The function of GF9 on sepsis was first discovered in 2014 (Sigalov, 2014). The administration of GF9 suppressed the TREM-1-mediated production of proinflammatory cytokines of TNF-α, IL-1B, and IL-6 *in vitro* (LPS) and *in vivo* (CLP) (Sigalov, 2014). In 2019, Gibot et al. (2019) confirmed the role of GF9 on sepsis, which was consistent with that reported by Sigalov (2014). In our present study, the nanomedicine of GF9-HFn significantly reduced the expression level of proinflammatory cytokines of TNF-α and IL-6, which agreed with the studies mentioned above (Sigalov, 2014; Gibot et al., 2019). TNF-α is accumulated in many pathological conditions, including sepsis, malignant tumors, heart failure, and chronic inflammatory diseases. IL-6 can make B cell precursors produce antibodies and induce inflammation in cells. Therefore, TNF-α and IL-6 are recognized as proinflammatory cytokines for sepsis. The decreased levels of TNF-α and IL-6 revealed the advantage therapy role of GF9 on sepsis. More importantly, the co-delivery of GF9 and streptomycin better reduces proinflammatory cytokines than single GF9.

Streptomycin is also one treatment of *E. coli*. In the study of Zhang et al. (2013), they demonstrated that streptomycin combined with kanamycin promotes the conjugation of *E. coli*. Therefore, we assumed that streptomycin was helpful in the treatment of *E. coli*-induced sepsis. However, the role of nanoparticles delivered GF9 combined with streptomycin on sepsis had never been discovered. Our study demonstrated that combination injection of nanomedicine GF9 and streptomycin have a better effect on overall survival rate, anti-inflammatory, and anti-bacterial in HUVEC and Gram-negative bacteria-induced sepsis mice. Moreover, the combined injection released the injury caused by sepsis. Thus, our present study revealed a new potential therapeutic method for sepsis.

In the present study, we successfully constructed a ferritin-based co-delivery nanomedicine system with molecules of GF9 and streptomycin. This nano drug can improve the overall survival rate of sepsis caused by Gram-negative bacteria and inhibit inflammatory factor (TNF-α And IL-6) and inhibit *E. coli*. This study provides a new idea and nano drug delivery method for the treatment of sepsis.

## Data Availability

The original contributions presented in the study are included in the article/supplementary material, further inquiries can be directed to the corresponding author.

## References

[B1] Bellini M. Riva B. Tinelli V. Rizzuto M. A. Salvioni L. Colombo M. (2020). Engineered ferritin nanoparticles for the bioluminescence tracking of nanodrug delivery in cancer. Small 16, e2001450. 10.1002/smll.202001450 32856404

[B2] Dantas P. H. d. S. Matos A. d. O. da Silva Filho E. Silva-Sales M. Sales-Campos H. (2020). Triggering receptor expressed on myeloid cells-1 (TREM-1) as a therapeutic target in infectious and noninfectious disease: a critical review. Int. Rev. Immunol. 39, 188–202. 10.1080/08830185.2020.1762597 32379561

[B3] Denning N.-L. Aziz M. Murao A. Gurien S. D. Ochani M. Prince J. M. (2020). Extracellular CIRP as an endogenous TREM-1 ligand to fuel inflammation in sepsis. Jci Insight 5, 134172. 10.1172/jci.insight.134172 32027618 PMC7141396

[B4] Fang Y. Gao F. Liu Z. (2019). Angiotensin-converting enzyme 2 attenuates inflammatory response and oxidative stress in hyperoxic lung injury by regulating NF-κB and Nrf2 pathways. QJM Int. J. Med. 112, 914–924. 10.1093/qjmed/hcz206 31393582

[B5] Gibot S. Jolly L. Lemarie J. Carrasco K. Derive M. Boufenzer A. (2019). Triggering receptor expressed on myeloid cells-1 inhibitor targeted to endothelium decreases cell activation. Front. Immunol. 10, 2314. 10.3389/fimmu.2019.02314 31632399 PMC6779727

[B6] Gyawali B. Ramakrishna K. Dhamoon A. S. (2019). Sepsis: The evolution in definition, pathophysiology, and management. SAGE open Med. 7, 2050312119835043. 10.1177/2050312119835043 30915218 PMC6429642

[B7] Ibraheem F. Aziz M. H. Fatima M. Shaheen F. Ali S. M. Huang Q. (2019). *In vitro* cytotoxicity, MMP and ROS activity of green synthesized nickel oxide nanoparticles using extract of Terminalia chebula against MCF-7 cells. Mater. Lett. 234, 129–133. 10.1016/j.matlet.2018.09.075

[B8] Inoue I. Chiba M. Ito K. Okamatsu Y. Suga Y. Kitahara Y. (2021). One-step construction of ferritin encapsulation drugs for cancer chemotherapy. Nanoscale 13, 1875–1883. 10.1039/d0nr04019c 33439183

[B9] Khoshnejad M. Parhiz H. Shuvaev V. V. Dmochowski I. J. Muzykantov V. R. (2018). Ferritin-based drug delivery systems: hybrid nanocarriers for vascular immunotargeting. J. Control. Release 282, 13–24. 10.1016/j.jconrel.2018.02.042 29522833 PMC6008199

[B10] Kim Y. I. Kim D. Yu K. M. Seo H. D. Lee S. A. Casel M. A. B. (2021). Development of spike receptor-binding domain nanoparticles as a vaccine candidate against SARS-CoV-2 infection in ferrets. mBio 12, e00230–00221. 10.1128/mBio.00230-21 33653891 PMC8092224

[B11] Kolhe P. Misra E. Kannan R. M. Kannan S. Lieh-Lai M. (2003). Drug complexation, *in vitro* release and cellular entry of dendrimers and hyperbranched polymers. Int. J. Pharm. 259, 143–160. 10.1016/s0378-5173(03)00225-4 12787643

[B12] Lee N. K. Lee E. J. Kim S. Nam G. H. Kih M. Hong Y. (2017). Ferritin nanocage with intrinsically disordered proteins and affibody: a platform for tumor targeting with extended pharmacokinetics. J. Control. Release 267, 172–180. 10.1016/j.jconrel.2017.08.014 28821462

[B13] Lee W. Seo J. Kwak S. Park E. J. Na D. H. Kim S. (2015). A double-chambered protein nanocage loaded with thrombin receptor agonist peptide (TRAP) and γ-carboxyglutamic acid of protein C (PC-Gla) for sepsis treatment. Adv. Mat. 27, 6637–6643. 10.1002/adma.201503093 26414883

[B14] Li B. Dou Z. Zhang L. Zhu L. Cao Y. Yu Q. (2021a). Ghrelin alleviates intestinal dysfunction in sepsis through the KLF4/MMP2 regulatory Axis by activating SIRT1. Front. Immunol. 12, 646775. 10.3389/fimmu.2021.646775 33968038 PMC8102724

[B15] Li J. Zhang Z. Deng H. Zheng Z. (2021b). Cinobufagin-loaded and folic acid-modified polydopamine nanomedicine combined with photothermal therapy for the treatment of lung cancer. Front. Chem. 9, 637754. 10.3389/fchem.2021.637754 33855009 PMC8039290

[B16] Li Y. Wang X. Yan J. Liu Y. Yang R. Pan D. (2019). Nanoparticle ferritin-bound erastin and rapamycin: a nanodrug combining autophagy and ferroptosis for anticancer therapy. Biomater. Sci. 7, 3779–3787. 10.1039/c9bm00653b 31211307

[B17] Liang Q. Zhou L. Li Y. Liu J. Liu Y. (2022). Nano drug delivery system reconstruct tumour vasculature for the tumour vascular normalisation. J. Drug Target. 30, 119–130. 10.1080/1061186X.2021.1927056 33960252

[B18] Lippert P. C. Zachos J. C. (2007). A biogenic origin for anomalous fine-grained magnetic material at the Paleocene-Eocene boundary at Wilson Lake, New Jersey. Paleoceanography 22, PA4104. 10.1029/2007PA001471

[B19] Livak K. J. Schmittgen T. D. (2001). Analysis of relative gene expression data using real-time quantitative PCR and the 2−ΔΔCT method. Methods 25, 402–408. 10.1006/meth.2001.1262 11846609

[B20] Meng J. B. Jiao Y. N. Zhang G. Xu X. J. Ji C. L. Hu M. H. (2018). Electroacupuncture improves intestinal dysfunction in septic patients: a randomised controlled trial. Biomed. Res. Int. 2018, 8293594. 10.1155/2018/8293594 30046610 PMC6038666

[B21] Pandolfi L. Bellini M. Vanna R. Morasso C. Zago A. Carcano S. (2017). H-ferritin enriches the curcumin uptake and improves the therapeutic efficacy in triple negative breast cancer cells. Biomacromolecules 18, 3318–3330. 10.1021/acs.biomac.7b00974 28886247

[B22] Partikel K. Korte R. Mulac D. Humpf H.-U. Langer K. (2019). Serum type and concentration both affect the protein-corona composition of PLGA nanoparticles. Beilstein J. Nanotechnol. 10, 1002–1015. 10.3762/bjnano.10.101 31165027 PMC6541368

[B23] Peng A. Lu X. Huang J. He M. Xu J. Huang H. (2019). Rheumatoid arthritis synovial fibroblasts promote TREM-1 expression in monocytes via COX-2/PGE 2 pathway. Arthritis Res. Ther. 21, 169. 10.1186/s13075-019-1954-3 31287012 PMC6615166

[B24] Saurer L. Zysset D. Rihs S. Mager L. Gusberti M. Simillion C. (2017). TREM-1 promotes intestinal tumorigenesis. Sci. Rep. 7, 14870. 10.1038/s41598-017-14516-4 29093489 PMC5665947

[B25] Sigalov A. B. (2014). A novel ligand-independent peptide inhibitor of TREM-1 suppresses tumor growth in human lung cancer xenografts and prolongs survival of mice with lipopolysaccharide-induced septic shock. Int. Immunopharmacol. 21, 208–219. 10.1016/j.intimp.2014.05.001 24836682 PMC4088342

[B26] Stanley S. (2014). Biological nanoparticles and their influence on organisms. Curr. Opin. Biotechnol. 28, 69–74. 10.1016/j.copbio.2013.11.014 24832077

[B27] Su L. Han B. Liu C. Liang L. Jiang Z. Deng J. (2012). Value of soluble TREM-1, procalcitonin, and C-reactive protein serum levels as biomarkers for detecting bacteremia among sepsis patients with new fever in intensive care units: a prospective cohort study. BMC Infect. Dis. 12, 157. 10.1186/1471-2334-12-157 22809118 PMC3426475

[B28] Xu Y. Zhang J. Liu X. Huo P. Zhang Y. Chen H. (2019). MMP-2-responsive gelatin nanoparticles for synergistic tumor therapy. Pharm. Dev. Technol. 24, 1002–1013. 10.1080/10837450.2019.1621899 31109231

[B29] Zhang P. Y. Xu P. P. Xia Z. J. Wang J. Xiong J. Li Y. Z. (2013). Combined treatment with the antibiotics kanamycin and streptomycin promotes the conjugation of *Escherichia coli* . FEMS Microbiol. Lett. 348, 149–156. 10.1111/1574-6968.12282 24111668

[B30] Zhao T. Pan B. Alam H. B. Liu B. Bronson R. T. Deng Q. (2016). Protective effect of Cl-amidine against CLP-induced lethal septic shock in mice. Sci. Rep. 6, 36696. 10.1038/srep36696 27819302 PMC5098180

[B31] Zhen Z. Tang W. Chen H. Lin X. Todd T. Wang G. (2013). RGD-modified apoferritin nanoparticles for efficient drug delivery to tumors. ACS Nano 7, 4830–4837. 10.1021/nn305791q 23718215 PMC3705644

[B32] Zhou Q. Zhang L. Yang T. Wu H. (2018). Stimuli-responsive polymeric micelles for drug delivery and cancer therapy. Int. J. Nanomedicine 13, 2921–2942. 10.2147/IJN.S158696 29849457 PMC5965378

[B33] Zou H. Jia X. He X. Su Y. Zhou L. Shen Y. (2021a). Emerging threat of multidrug resistant pathogens from neonatal sepsis. Front. Cell. Infect. Microbiol. 11, 694093. 10.3389/fcimb.2021.694093 34322398 PMC8312093

[B34] Zou T. Lu W. Mezhuev Y. Lan M. Li L. Liu F. (2021b). A review of nanoparticle drug delivery systems responsive to endogenous breast cancer microenvironment. Eur. J. Pharm. Biopharm. 166, 30–43. 10.1016/j.ejpb.2021.05.029 34098073

