# Corrigendum: Synergistic effect of GF9 and streptomycin on relieving gram-negative bacteria-induced sepsis

**DOI:** 10.3389/fbioe.2023.1204254

**Published:** 2023-06-16

**Authors:** Bing Wei, Yingmin Ma

**Affiliations:** ^1^ Emergency Medicine Clinical Research Center, Beijing Chao-Yang Hospital, Capital Medical University, Beijing, China; ^2^ Beijing Key Laboratory of Cardiopulmonary Cerebral Resuscitation, Clinical Center for Medicine in Acute Infection, Capital Medical University, Beijing, China; ^3^ Department of Respiratory and Critical Care Medicine, Beijing Youan Hospital, Capital Medical University, Beijing, China

**Keywords:** nanomedicine, GF9, streptomycin, sepsis, gram-negative bacteria-induced

In the published article, there was an error. The molecular weights of “GF9-HFn/Str, HFn, GF9-HFn and GF9-HFn + MMP2” in the original article were wrong. The molecular weights of “GF9-HFn/Str, HFn and GF9-HFn” in the original article were displayed as “36 kD, 33.1 kD and 36 kD”. But the correct size of “GF9-HFn/Str is 40 kD”, the correct size of “HFn is 35 kD” and the correct size of “GF9-HFn is 40 kD”.

In the published article, there was an error in **Abstract section**.

“The size of GF9-HFn/Str is 36 kD”.

The corrected sentence appears below:

“The size of GF9-HFn/Str is 40 kD”.

In the published article, there was an error in **Results section** titled *The evaluation of ferritin-based codelivery nanoparticle*.

“We found that the size of GF9-HFn/Str (36 kD) is bigger than HFn (33.1 kD; **Figure 1A**)”.

The corrected sentence appears below:

“We found that the size of GF9-HFn/Str (40 kD) is bigger than HFn (35 kD; **Figure 1A**)”.

In the published article, there was an error in **Results section** titled *GF9 can be released by MMP2*.

“Then, SDS-PAGE assessed the protein molecules. Compared with GF9-HFn (36 kD), the cells added MMP2 enzyme separated the band of GF9-HFn + MMP2 (36 kD) into two bands, which are HFn (33.1 kD) and GF9 (2.9 kD) (**Figure 2B**)”.

The corrected sentence appears below:

“Then, SDS-PAGE assessed the protein molecules. Compared with GF9-HFn (40 kD), the cells added MMP2 enzyme separated the band of GF9-HFn + MMP2 into two bands, which are HFn (35 kD) and GF9 (2.9 kD) (**Figure 2B**)”.

The authors apologize for this error and state that this does not change the scientific conclusions of the article in any way. The original article has been updated.

